# Comparative Pharmacovigilance Analysis of Selected Cardiotoxicity Signals Associated with Doxorubicin and Epirubicin Based on EudraVigilance Data

**DOI:** 10.3390/jcm15176734

**Published:** 2026-08-30

**Authors:** Emilia Sorina Fiat, Anca Butuca, Carmen Maximiliana Dobrea, Steliana Ghibu, Razvan Constantin Vonica, Adina Frum, Claudiu Morgovan, Nastaca Alina Palade, Crina Cristina Solomon, Florina Batar, Bogdan Ioan Vintila, Maria Totan, Felicia Gabriela Gligor

**Affiliations:** 1 Faculty of Medicine, “Lucian Blaga” University of Sibiu, 550169 Sibiu, Romania; sorina.fiat@yahoo.com (E.S.F.); razvanconstantin.vonica@ulbsibiu.ro (R.C.V.); adina.frum@ulbsibiu.ro (A.F.); claudiu.morgovan@ulbsibiu.ro (C.M.); nastacaalina.coman@ulbsibiu.ro (N.A.P.); crinacristina.solomon@ulbsibiu.ro (C.C.S.); florina.batar@ulbsibiu.ro (F.B.); bogdan.vintila@ulbsibiu.ro (B.I.V.); maria.totan@ulbsibiu.ro (M.T.); felicia.gligor@ulbsibiu.ro (F.G.G.); 2 Department of Cardiology, County Clinical Emergency Hospital, 550245 Sibiu, Romania; 3 Faculty of Pharmacy, “Iuliu Hatieganu” University of Medicine and Pharmacy, 6A Louis Pasteur Street, 400349 Cluj-Napoca, Romania; steliana.ghibu@umfcluj.ro

**Keywords:** anthracyclines, cardiotoxicity, cardio-oncology, pharmacovigilance, adverse drug reactions, EudraVigilance

## Abstract

**Background/Objectives:** Anthracycline-induced cardiotoxicity may compromise cancer treatment and long-term outcomes. Although differences in the cardiotoxicity profile of epirubicin and doxorubicin have been reported, direct comparative pharmacovigilance evidence remains limited. This study compared their cardiovascular adverse reaction reporting profiles using EudraVigilance (EV) data. **Methods:** Aggregated Individual Case Safety Reports submitted up to 12 July 2026 on the European portal were analyzed. Descriptive analyses assessed demographic characteristics, report origin, reporter type, System Organ Class distribution, seriousness, and clinical outcomes. Cardiotoxicity-related preferred terms (PTs) were identified using the Standardized MedDRA Queries “Cardiac failure”, “Cardiomyopathy”, and “Myocardial infarction”. Comparative disproportionality analysis was restricted to reports submitted by healthcare professionals. Reporting odds ratios and 95% confidence intervals were calculated for PTs with at least five reports for each drug. **Results:** Overall, 52,257 reports for doxorubicin and 31,049 for epirubicin were identified. Cardiac disorders were reported in 4459 doxorubicin cases and 1406 epirubicin cases, with over 97% classified as serious. Among 51 cardiotoxicity-related PTs, doxorubicin showed significantly higher reporting odds for cardiogenic shock, congestive, chronic, acute, and left ventricular cardiac failure, decreased ejection fraction, cardiomyopathy, cardiotoxicity, toxic cardiomyopathy, acute myocardial infarction, myocardial infarction, and increased troponin. The strongest disproportionality signals were observed for cardiogenic shock, toxic cardiomyopathy, cardiomyopathy, and cardiotoxicity. **Conclusions:** Doxorubicin was associated with a more pronounced pattern of cardiotoxicity-related reporting and disproportionality signals than epirubicin in the EV database. These findings highlight potential differences in the cardiovascular safety profiles of the two anthracyclines and provide signals that warrant further investigation, while underscoring the importance of cardiovascular risk assessment and monitoring in patients receiving anthracycline therapy. However, disproportionality signals do not establish incidence, absolute risk, or causality and require confirmation in prospective comparative studies.

## 1. Introduction

Anthracycline treatment represents an essential therapeutic component for malignant tumors, particularly breast cancer, lymphomas, leukemias, sarcomas, and other solid and hematological malignancies. Doxorubicin and epirubicin are cytostatics used in both curative and palliative treatments due to their broad antitumor mechanism and well-established clinical efficacy [1]. Regarding their mechanism of action, anthracyclines intercalate between DNA bases and stabilize the DNA–topoisomerase II complex, preventing strand re-ligation and causing double-strand breaks, replication blockade, and cell death. In parallel, they produce reactive oxygen species and mitochondrial dysfunction, mechanisms that contribute to both their antitumor effect and their cumulative cardiotoxicity [2]. Cardiac toxicity can compromise treatment administration, increasing the risk of non-cancer morbidity and mortality among cancer survivors, and can also affect long-term quality of life. Anthracycline-induced cardiotoxicity remains one of the primary clinical challenges in contemporary cardio-oncology [3].

Cardiovascular manifestations associated with anthracyclines comprise a heterogeneous group of symptoms. Initially, cardiac dysfunction may be asymptomatic, associated with a decrease in left ventricular ejection fraction or an increase in cardiac biomarkers. In other cases, myocardial injury can progress to symptomatic heart failure or dilated cardiomyopathy [4]. Acute manifestations, including arrhythmias, electrocardiographic abnormalities, pericardial involvement, and transient ventricular dysfunction, can occur during or shortly after treatment, whereas chronic toxicity may become clinically apparent months or years after completing chemotherapy [5].

Cumulative exposure to anthracycline treatment is directly proportional to cardiac risk; however, toxicity can also occur at relatively low doses, especially in patients with pre-existing cardiac diseases, arterial hypertension, diabetes mellitus, advanced age, or a history of prior thoracic radiation therapy exposure. Additionally, exposure to other cardiotoxic treatments can increase the risk [6].

From a physiopathological point of view, anthracycline-induced myocardial lesions result from the complex interaction of molecular and cellular mechanisms [7]. Redox cycling and the production of reactive oxygen species are considered the central mechanisms, particularly for cardiomyocytes, which have a high mitochondrial density and a more limited antioxidant capacity [8]. The inhibition of topoisomerase IIβ in cardiomyocytes contributes to DNA double-strand breaks, impaired mitochondrial biogenesis, oxidative stress, and progressive ventricular dysfunction [9,10]. Other supplementary processes are also mentioned, such as lipid peroxidation, ferroptosis, calcium overload, inflammation, altered autophagy, endothelial dysfunction, and cellular senescence, which can amplify or perpetuate myocardial injury [11]. Consequently, these mechanisms can lead to the cumulative loss of functional cardiomyocytes and a reduction in myocardial reserve [12]. In certain situations, these mechanisms can explain clinical manifestations that may appear long after the initial exposure to anthracyclines.

The cardiotoxicity of anthracyclines varies, even though they belong to the same pharmacological class. Epirubicin is associated with lower cardiovascular toxicity compared to doxorubicin, especially when conventional cumulative doses are considered [5]. However, direct comparisons are difficult because the two agents are administered at different dose intensities and are frequently combined with various cytotoxic, targeted, or radiation-based treatments [13].

A cardio-oncological evaluation is recommended to assess cardiovascular risk prior to initiating anthracycline treatment, followed by surveillance tailored to the patient’s initial risk as well as cumulative anthracycline exposure [14]. Electrocardiography, transthoracic echocardiography—including left ventricular ejection fraction—and, when available, the measurement of cardiac troponins and natriuretic peptides are the primary tools for detecting early myocardial injury.

Recognizing symptomatology or identifying cardiotoxicity in a timely manner is clinically relevant, as the probability of functional recovery is higher when heart failure therapy is initiated shortly after cardiac dysfunction becomes apparent [15].

The present study aimed to characterize and compare selected cardiotoxicity-related adverse drug reactions (ADRs) associated with doxorubicin and epirubicin using Individual Case Safety Reports (ICSRs) submitted to the EudraVigilance database. Specifically, disproportionality analyses were performed for preferred terms (PTs) included in the Standardized MedDRA Queries (SMQs) “Cardiac failure”, “Cardiomyopathy”, and “Myocardial infarction” to identify differences in reporting patterns between the two anthracyclines. The findings provide pharmacovigilance evidence that complements existing clinical and mechanistic knowledge regarding their relative cardiovascular safety profiles.

## 2. Materials and Methods

### 2.1. Study Design

A pharmacovigilance study on ADRs reported in the EudraVigilance (EV) database for two anthracyclines, doxorubicin and epirubicin, was conducted. Aggregated data from ICSRs submitted until 12 July 2026 on the European portal https://www.adrreports.eu/ (accessed on 14 July 2026) were used. Using data extracted, descriptive and disproportionality analyses were performed. ICSRs could be filled by healthcare professionals (HP) or Non-HP, for patients originating from the European Economic Area (EEA) or Non-EEA [16].

#### Inclusion and Exclusion Criteria

Doxorubicin and epirubicin were selected because of their close structural similarity; epirubicin being a structural analog of doxorubicin that was widely regarded as a potentially less cardiotoxic alternative, making this comparison of particular clinical relevance. EV aggregate data does not allow separate identification of reports for liposomal doxorubicin and conventional doxorubicin. Consequently, liposomal doxorubicin was not analyzed as a separate entity. Furthermore, daunorubicin, idarubicin, and mitoxantrone were excluded to reduce clinical heterogeneity, as these agents differ from doxorubicin and epirubicin with respect to their predominant therapeutic indications, treatment settings, and safety profiles. No reports for amrubicin and valrubicin were identified in the EudraVigilance database; therefore, it was not included in the analysis [17]. The minimum threshold of five reports was applied after restricting the dataset to HP reports.

### 2.2. Material

According to the European Medicines Agency (EMA) regulations, different PTs categorized under 27 System Organ Classes (SOCs) can be used to report ADRs [18]. A SOC represents the highest level of hierarchy, and PTs represent a medical terminology that defines a unique adverse effect or clinical condition reported in the database. The Medical Dictionary for Regulatory Activities (MedDRA) provides a hierarchical structure for different categories of terms classified as “medical and health-related”. It also includes PTs, which are standardized medical terms used for coding ADRs. In the present study, PTs were identified from the corresponding Standardized MedDRA Queries (SMQs), which comprise predefined groupings of MedDRA terms related to specific safety topics [19]. To evaluate anthracycline cardiotoxicity, three SMQs were selected (“Cardiac failure”, “Myocardial infarction” and “Cardiomyopathy”). These SMQs were selected because they capture the main clinically relevant manifestations of anthracycline-associated cardiotoxicity described in the literature [15,20]. SMQs were used to identify relevant PTs (n = 51) related to cardiotoxicity, and disproportionality analysis was conducted at the individual PT level (Supplementary Material Table S1).

### 2.3. Data Analysis

#### 2.3.1. Descriptive Analysis

A comparative analysis based on general characteristics reported for doxorubicin and epirubicin was performed: (i) patients’ age groups (0–1 month, 2 months–2 years, 3–11 years, 12–17 years, 18–64 years, 65–85 years, over 85 years, or not specified); (ii) sex (male, female, or not specified); (iii) geographic origin (EEA, non-EEA, or not specified); (iv) reporter category (HP, non-HP, or not specified) [21]. The distribution of cases by SOC reported for both anthracyclines was analyzed, and subsequently the distribution of cases by seriousness (serious, non-serious, or not specified) was compared across doxorubicin and epirubicin. A serious adverse reaction was considered if it resulted in death, persistent or significant disability or incapacity, or a birth defect; was life-threatening; or required hospitalization or prolongation of existing hospitalization [22,23]. For each SOC, the proportion of serious reports was calculated by dividing the number of serious reports by the total number of reports within the corresponding SOC and expressing it as a percentage. Subsequently, a Cleveland dot plot was used to compare these percentages between the two anthracyclines across SOCs. To facilitate visual comparison, SOCs were ordered according to the absolute difference in the proportion of serious reports between doxorubicin and epirubicin.

To identify similarities and differences in outcome patterns across PTs and between anthracyclines and to facilitate the visual comparison of clinical outcomes, three heatmaps were generated separately for each SMQ related to anthracycline cardiotoxicity. The frequency of each clinical outcome category of each PT was calculated separately for epirubicin and doxorubicin. These frequencies were normalized to the total number of reports for the corresponding PT and drug. Outcome frequencies were expressed as percentages, allowing comparison of outcome distributions independent of the absolute number of reports. Heatmaps were constructed using identical outcome categories for both drugs: Fatal, Not recovered/Not resolved, Not specified, Recovered/Resolved, Recovered/Resolved with sequelae, Recovering/Resolving, and Unknown. The color intensity represents the relative proportion of reports within each PT.

#### 2.3.2. Disproportionality Analysis

Disproportionality analysis was performed to evaluate differences in reporting frequencies between the two anthracyclines. Based on the reporting odds ratio (ROR) and 95% confidence intervals (CI), similarities and differences in ADR reporting could be evaluated. If at least five cases were reported for each PT, the ROR was calculated by comparing the reporting odds of each ADR between the two drugs. For each PT, a 2 × 2 contingency table was constructed using HP reports only, where **a** represented the number of reports of the PT associated with doxorubicin, **b** the number of reports of all other PTs associated with doxorubicin, **c** the number of reports of the same PT associated with epirubicin, and **d** the number of reports of all other PTs associated with epirubicin. The ROR was calculated as:

$$ROR=\frac{a\times d}{b\times c}$$

The minimum threshold of five reports was applied after restricting the dataset to HP reports.

The ROR is a measure of disproportionality in spontaneous reporting databases and should be interpreted as a signal detection metric rather than a measure of comparative risk or causality. A statistically significant disproportionality signal was considered present when the lower limit of the 95% CI exceeded 1 [24,25,26]. However, the analysis was restricted to ICSRs submitted by HP, thereby excluding consumer reports [27]. To assess the robustness of the findings, a sensitivity analysis was additionally performed using the overall EV dataset, including reports submitted by HP, non-HP, and not-specified reports. The same disproportionality analysis and signal detection criteria were applied.

### 2.4. Ethics

The present study was based exclusively on anonymized pharmacovigilance data and did not involve access to any personally identifiable information. Consequently, ethics committee approval and informed consent were not required [27].

## 3. Results

### 3.1. Descriptive Analysis

#### 3.1.1. Analysis of General Characteristics Included in ICSRs

Until 12 July 2026, 52,257 reports were submitted in the EV database for doxorubicin and 31,049 reports for epirubicin. Reports were submitted most frequently in the 18–64 years category (47.3% for doxorubicin and 76.8% for epirubicin). On the other hand, 24.6% of ICSRs were reported for doxorubicin and 16.9% for epirubicin in the 65–85 years category. The highest proportion of reports was registered in females (85.5% for epirubicin and 55.1% for doxorubicin) and in non-EEA countries (67.1% for doxorubicin and 80.1% for epirubicin). However, HPs filled the majority of ICSRs (95.3% for doxorubicin and 89.4% for epirubicin) (Table 1).

#### 3.1.2. Distribution of ADRs by SOC

Regarding the distribution of reported ADRs by SOC, a predominance of hematologic events could be observed for both anthracyclines (17,273 cases for doxorubicin and 16,050 cases for epirubicin). Also, a high number of reports included ADRs from the “General disorders and administration site conditions”, “Investigations”, and “Gastrointestinal disorders” SOC.

However, for doxorubicin, the “Infections and infestations” SOC (n = 9334) ranked third in frequency of reported ADRs, while for epirubicin, “Hepatobiliary disorders” SOC placed fifth (n = 1676) (Table 2).

#### 3.1.3. Distribution of Cases by Seriousness

In the majority of SOCs, more than 90% of cases were reported as serious (Table 3). For doxorubicin, non-serious reported ADRs were related in the highest proportion to “Musculoskeletal and connective tissue disorders” (13.7%) and “Skin and subcutaneous tissue disorders” (13.7%). However, non-serious epirubicin cases were reported with the highest frequency in the following SOCs: “Ear and labyrinth disorders” (27.7%), “Reproductive system and breast disorders” (24.6%), “Skin and subcutaneous tissue disorders” (22.5%), “Eye disorders (22.2%)”, “Product issues” (20.0%), “Gastrointestinal disorders” (19.0%), and “Nervous system disorders” (18.4%).

**Figure 1:**
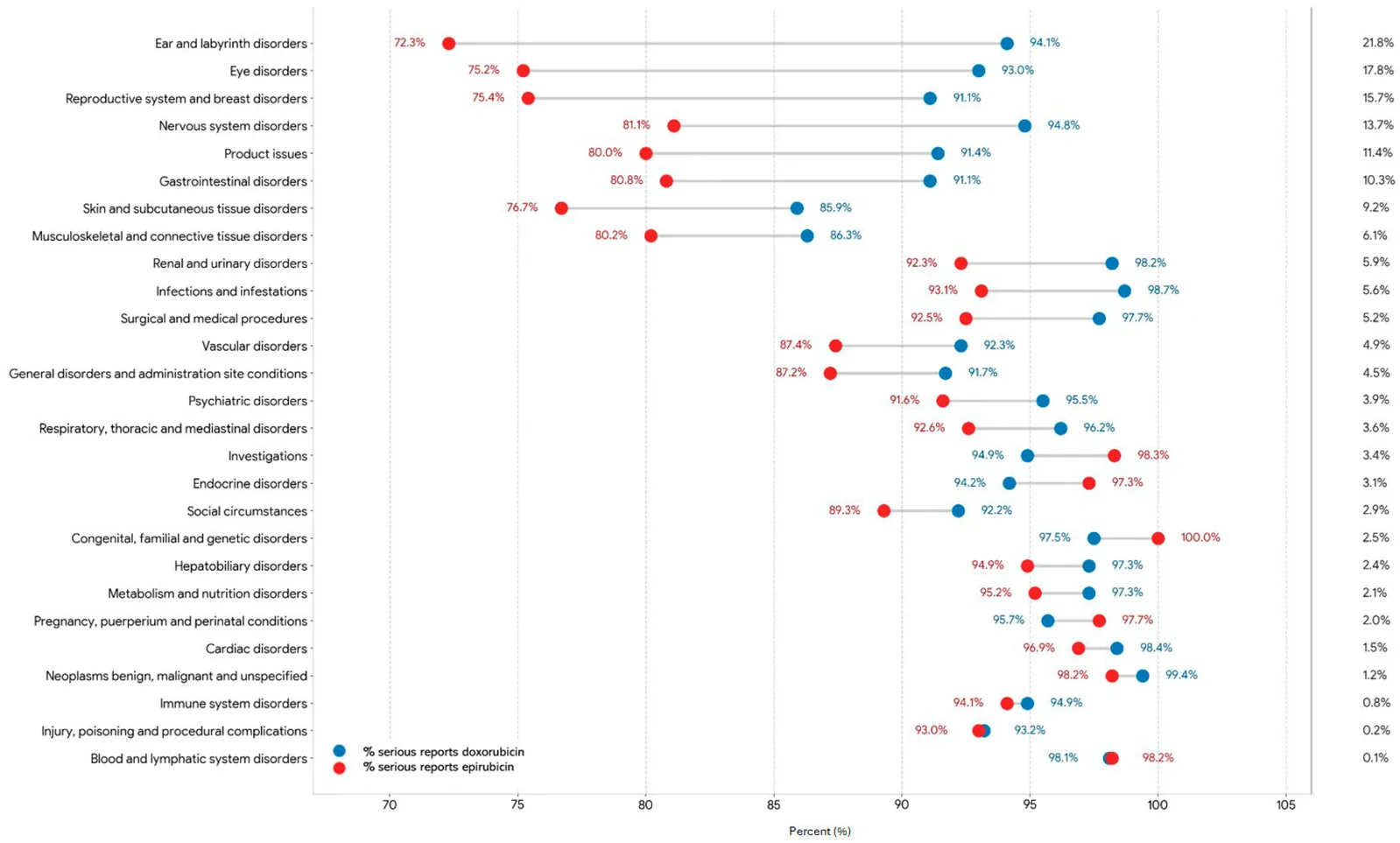
Distribution of serious reports across SOCs for doxorubicin and epirubicin. Points represent the proportion of serious reports within each SOC for each anthracycline. Gray lines connect corresponding SOCs, and SOCs are ordered by the absolute difference in the percentage of serious reports between the two drugs.

Generally, serious cases reported for doxorubicin had a higher frequency than for epirubicin. Thus, the largest differences between these proportions were observed for the following SOCs: “Ear and labyrinth disorders” (21.8%), “Eye disorders” (17.8%), “Reproductive system and breast disorders” (15.7%), “Nervous system disorders” (13.7%), and “Product issues” (11.4%). However, in five SOCs, serious cases reported for epirubicin had a higher frequency than for doxorubicin (“Investigations”, 3.4%; “Endocrine disorders”, 3.1%; “Congenital, familial and genetic disorders”, 2.5%; “Pregnancy, puerperium and perinatal conditions”, 2.1%; and “Blood and lymphatic system disorders, 0.1%) (Figure 1).

#### 3.1.4. Distribution of Cardiotoxicity-Related PTs

A total of 51 cardiotoxicity-related PTs included in three SMQs (“Cardiac Failure”, “Cardiomyopathy” and “Myocardial Infarction”) were identified among ICSRs associated with epirubicin and doxorubicin. Across both anthracyclines, the most frequently reported PTs were “Cardiac failure”, “Cardiomyopathy”, “Cardiotoxicity”, “Ejection fraction decreased”, and “Dilated cardiomyopathy”. However, for the majority of PTs, the absolute number of reports was higher for doxorubicin than for epirubicin (Supplementary Material—Table S2).

#### 3.1.5. Distribution of Clinical Outcomes Across Cardiotoxicity-Related Preferred Terms

**Figure 2:**
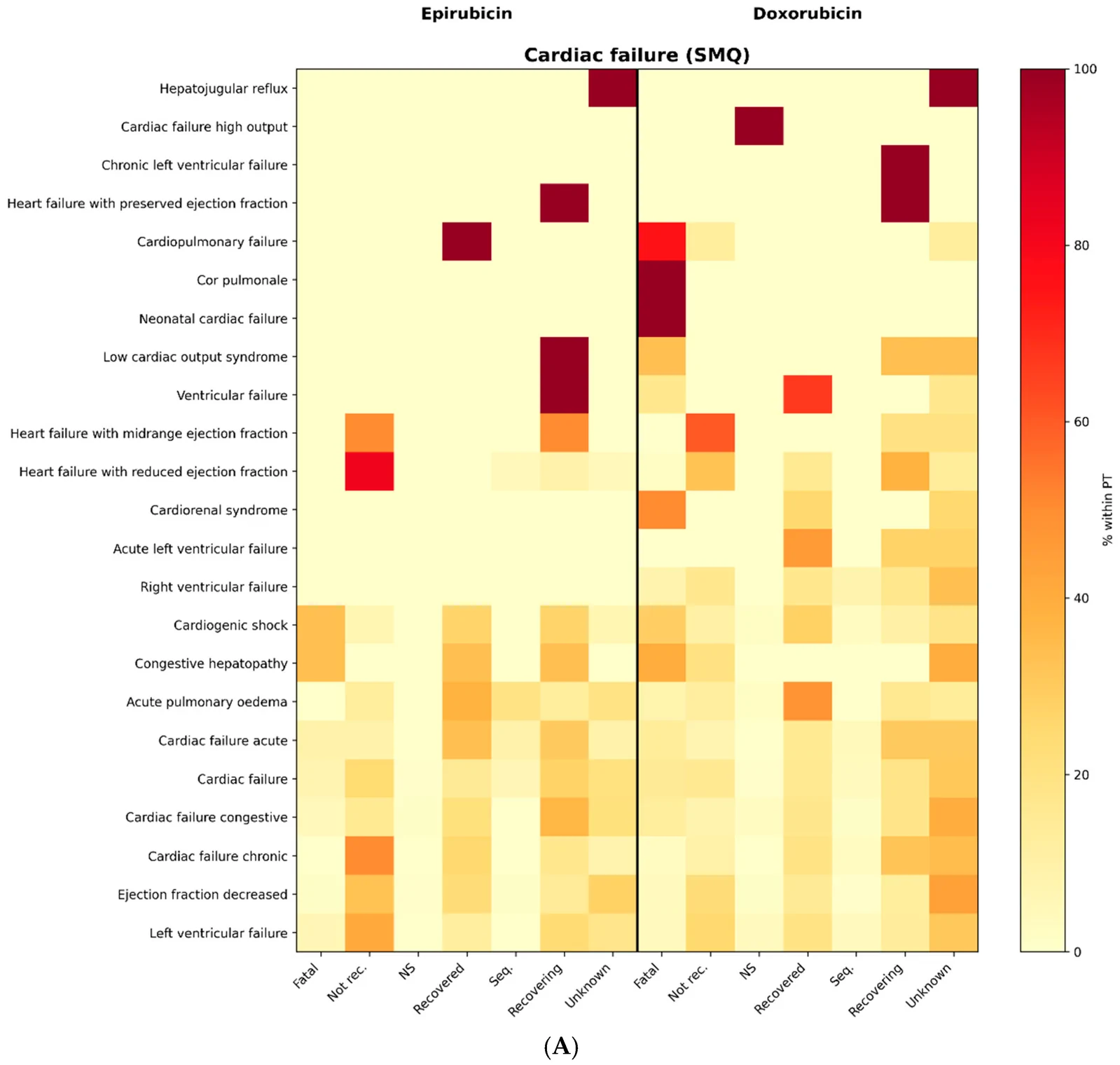

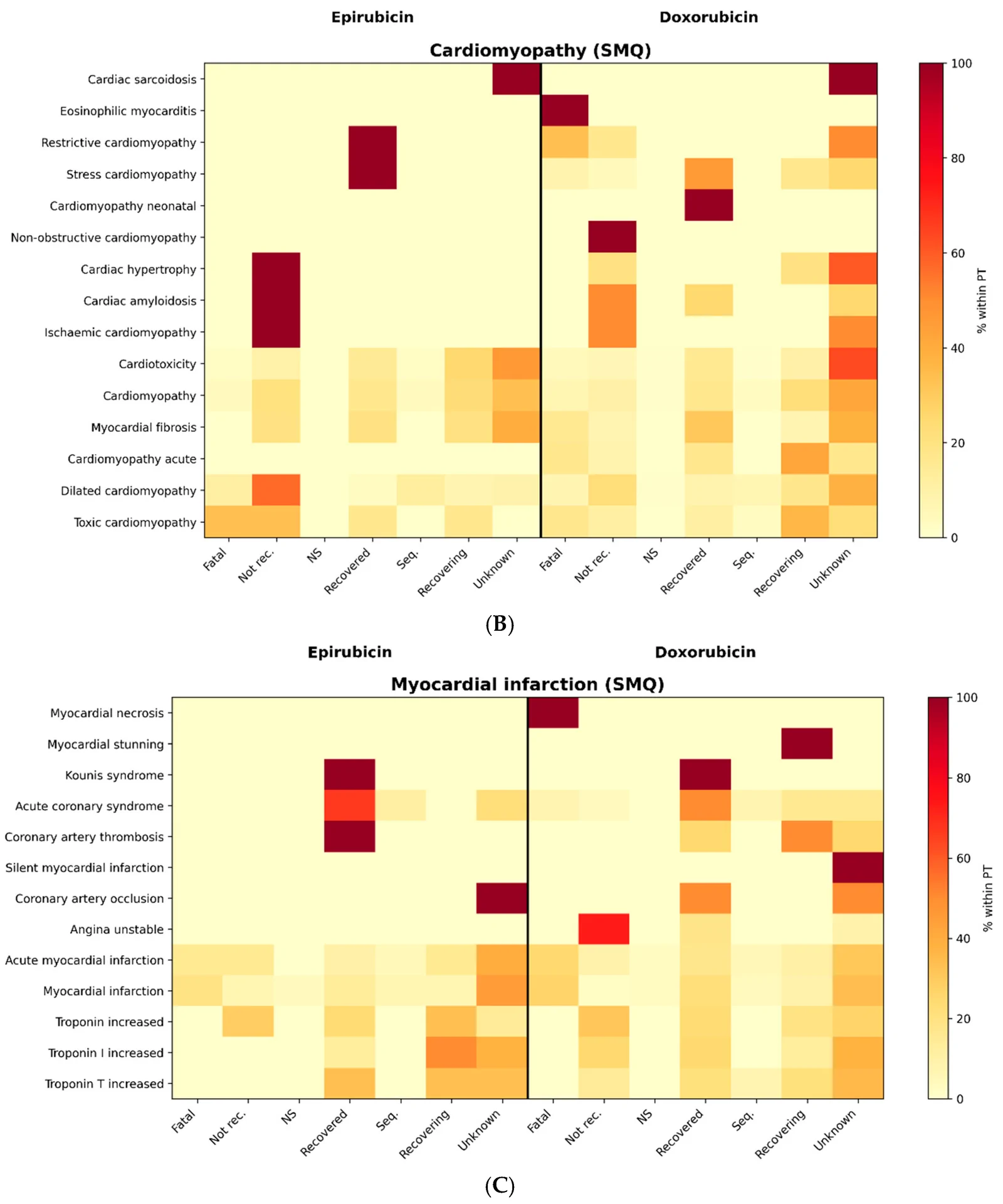
Heatmap analysis of clinical outcomes associated with cardiotoxicity-related Standardized MedDRA Queries (SMQs) following epirubicin and doxorubicin exposure. (**A**) Cardiac failure SMQ, (**B**) cardiomyopathy SMQ, and (**C**) myocardial infarction SMQ. Rows represent preferred terms (PTs), whereas columns represent clinical outcomes reported in EudraVigilance for epirubicin and doxorubicin. Outcome categories include: Fatal; Not rec.—Not recovered/Not resolved; NS—Not specified; Recovered—Recovered/Resolved; Seq.—Recovered/Resolved with sequelae; Recovering—Recovering/Resolving; and Unknown. For each PT, outcome frequencies were normalized to the total number of reports for the corresponding drug and expressed as percentages. Hierarchical clustering was applied to PTs to identify events with similar outcome distributions, while the order of outcome categories was kept identical for both drugs to facilitate direct comparison. Color intensity reflects the relative frequency of each outcome within a PT, with darker colors indicating higher proportions. Percentages for PTs with very small numbers of reports should be interpreted with caution, as normalization may exaggerate visual differences. Absolute numbers for each PT are provided in Supplementary Table S3.

For each PT and each drug, the frequency of every clinical outcome category was calculated separately. Heatmap analysis demonstrated heterogeneous distributions of clinical outcomes across PTs within each cardiotoxicity-related SMQ (Figure 2 and Supplementary Material—Table S3). Although several PTs shared similar outcome profiles, distinct differences were observed between epirubicin and doxorubicin for selected events. Overall, fatal and unresolved outcomes tended to predominate in severe manifestations such as cardiac failure and cardiogenic shock, whereas higher proportions of recovered or recovering outcomes were observed for less severe or reversible manifestations. For better visualization, the normalization of outcome frequencies within each PT was performed.

### 3.2. Disproportionality Analysis

**Figure 3:**
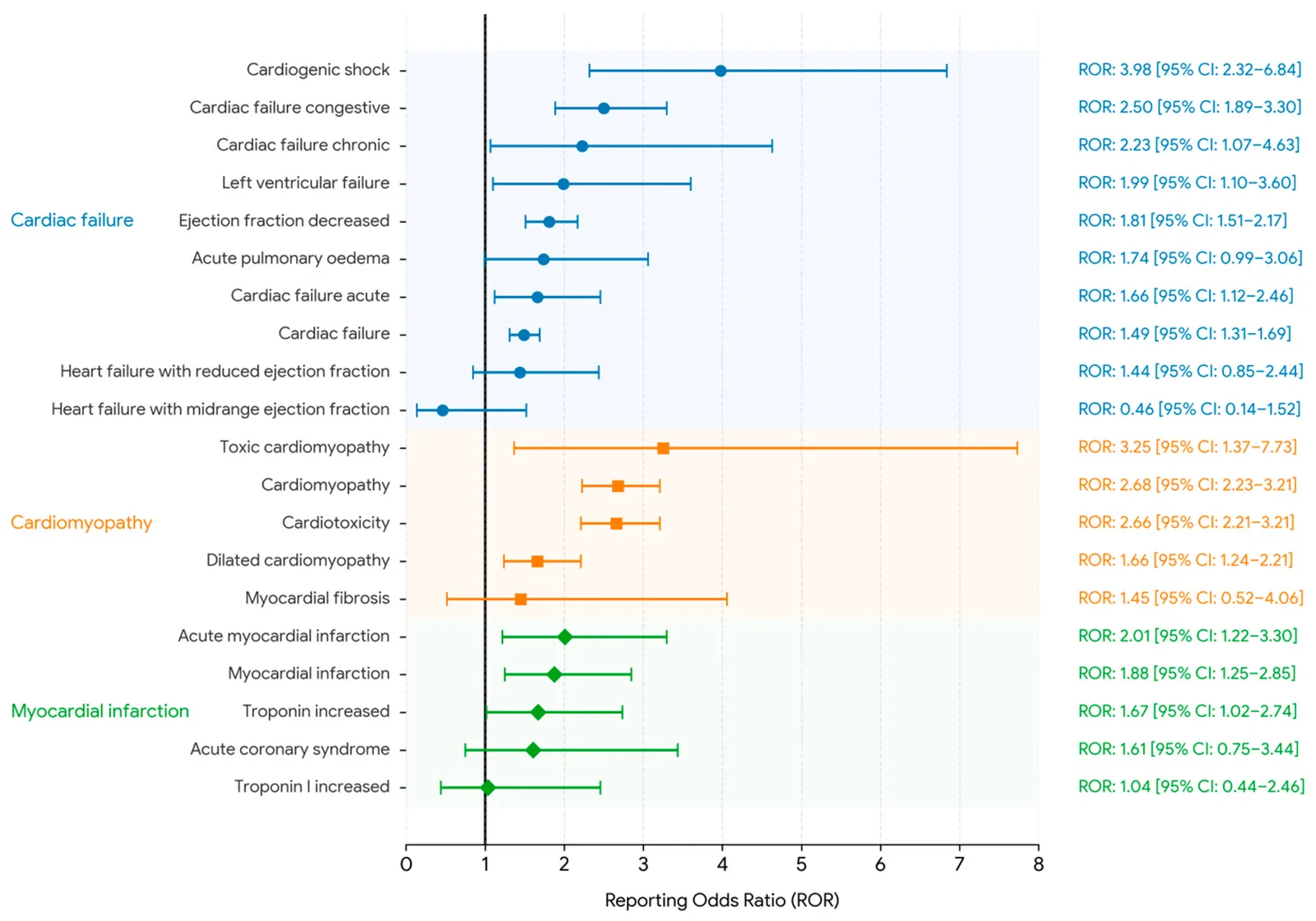
Disproportionality analysis for PTs of interest.

To improve the robustness of the analysis, the disproportionality evaluation was based exclusively on ICSRs submitted by HPs. A total of 51 PTs were identified across the three selected SMQs. Following application of the minimum report threshold, 20 PTs were eligible for disproportionality analysis. Within this dataset, doxorubicin demonstrated a higher reporting probability than epirubicin for the majority of PTs related to “Cardiac failure”, “Cardiomyopathy” and “Myocardial infarction”, suggesting a more pronounced cardiotoxic profile (Figure 3 and Supplementary Material—Table S4). Seven disproportionate signals were identified in “Cardiac failure” SMQ: “Cardiogenic shock” (ROR = 3.98; CI95%: 2.32–6.84), “Cardiac failure congestive” (ROR = 2.50; CI95%: 1.89–3.30), “Cardiac failure chronic” (ROR = 2.23; CI95%: 1.07–4.63), “Left ventricular failure” (ROR = 1.99; CI95%: 1.10–3.60), “Ejection fraction decreased” (ROR = 1.81; CI95%: 1.51–2.17), “Cardiac failure acute” (ROR = 1.66; CI95%: 1.12–2.46), and “Cardiac failure” (ROR = 1.49; CI95%: 1.31–1.69). No statistically significant differences between the two anthracyclines were noticed for “Acute pulmonary oedema”, “Heart failure with midrange ejection fraction” and “Heart failure with reduced ejection fraction”. In “Cardiomyopathy” SMQ, all PTs presented a significant disproportionality, except “Myocardial fibrosis”. The strongest signal was identified for “Toxic cardiomyopathy” (ROR = 3.25; CI95%: 1.37–7.73), “Cardiomyopathy” (ROR = 2.68; CI95%: 2.23–3.21), and “Cardiotoxicity” (ROR = 2.66; CI95%: 2.21–3.21). Regarding the PTs related to “Myocardial infarction” SMQ, doxorubicin presents a higher probability for “Acute myocardial infarction” (ROR = 2.01; CI95%: 1.22–3.30), “Myocardial infarction” (ROR = 1.88; CI95%: 1.25–2.85), and “Troponin increased” (ROR = 1.67; CI95%: 1.02–2.74). No significant differences were observed for “Acute coronary syndrome” and “Troponin I increased”. The sensitivity analysis based on the overall EV dataset yielded results that were largely consistent with those obtained using healthcare professional reports only (Supplementary Table S5). The only discrepancy was observed for the PT “Acute pulmonary oedema”, which met the disproportionality criterion in the overall dataset but not in the HP-only analysis. No other differences in signal detection were identified.

## 4. Discussion

Breast cancer is one of the leading malignancies in several countries. Continuous studies on the safety profile of medicines prescribed for this condition contribute to sustaining clinicians in making informed decisions when prescribing. Although cardiac toxicity of doxorubicin and epirubicin is known [28], this study compares drug-versus-drug AEs related to reported cardiac toxicity, while other recent studies used the general background of the entire pharmacovigilance database for comparison [13]. This approach offers a one-to-one comparison, and despite the limitations of pharmacovigilance database studies, it brings additional evidence regarding the reported cardiac adverse event profiles of doxorubicin and epirubicin.

The higher number of reports for doxorubicin may reflect its wider use, as demonstrated by the Summary of Product Characteristics (SmPC), doxorubicin has an almost triple number of distinct indications compared to epirubicin [29,30]. Furthermore, doxorubicin has a longer period of use in clinical practice, having been approved for marketing in the 1970s both in Europe and the United States of America [31]. Both elements, longer time and wider exposure, may contribute to a higher volume of reporting, without directly indicating a lower safety profile.

The highest proportion of reports for doxorubicin and epirubicin were in the adult population (>18 years). In both cases, female patients predominate, but the difference is much more pronounced for epirubicin (85.5%) compared to doxorubicin (55.1%). Epirubicin is predominantly reported in adult women aged 18 to 64 years, which is consistent with its main therapeutic indications, especially in breast cancer [29]. As expected, this finding confirms that female patients generated more reports, a situation also observed in the FDA Adverse Event Reporting System (FAERS) [32]. The small number of reports related to the male population should be interpreted carefully; they do not directly imply gender specific toxicity, rather they may reflect differences in the prevalence of malignancies for which these drugs are used and in the underlying population exposed to treatment; for example, breast cancer being diagnosed in men in much lower proportions than in women [33].

Most of the reports come from countries outside the European Economic Area (non-EEA). This distribution indicates that both drugs are reported globally, with a greater contribution from non-EEA countries, especially in the case of epirubicin. A high number of reports for both drugs have also been uploaded to FAERS [13].

The overwhelming majority of reports were submitted by HP, accounting for 95.3% for doxorubicin and 89.4% for epirubicin. This suggests that for the two molecules, the database is mainly powered by medical reports, which can contribute to a better quality of clinical information. Oncology patients are closely monitored by HP, highly trained personnel who can accurately identify ADRs and report them [34].

The first five SOCs differed between doxorubicin and epirubicin, although both drugs exhibit a predominance of reported hematological events. This pattern is consistent with the known toxicity profile of anthracyclines, characterized by myelosuppression and other hematological disorders [29,30]. A higher proportion of doxorubicin reports concerned infections, while epirubicin is noted for a higher proportion of reports classified as investigations and hepatobiliary disorders. These differences may reflect specificities of clinical use, patient monitoring, and reporting practices in the EV database, rather than differences in the comparative incidence or risk of these events. The results confirm the findings of Yang et al., who have analyzed the reports from FAERS [13].

In almost all SOC categories, serious reactions accounted for more than 90% of all reports, confirming the characteristic safety profile of anthracyclines and the context of their use in the treatment of oncological conditions [35]. High numbers of serious cases related to doxorubicin and epirubicin were also identified in VigiBase, the pharmacovigilance database of the World Health Organization; most reports for doxorubicin originated from Europe, while Asia leads in the epirubicin reports category [36].

The disproportionality analysis of the two medicines showed doxorubicin as having a higher probability of reporting cardiac failure, cardiogenic shock, decreased ejection fraction, left ventricular failure, cardiomyopathy, cardiotoxicity, dilated cardiomyopathy, toxic cardiomyopathy, acute myocardial infarction, myocardial infarction, and increased incidence than epirubicin.

The main finding shows that doxorubicin exhibits a greater disproportionality in reporting cardiotoxicity events compared to epirubicin. This is consistent with experimental studies [37], clinical trials, and experience gained in oncology practice, which have shown that epirubicin has a more favorable cardiac profile [38,39,40,41]. The consistency between the primary HP-only analysis and the sensitivity analysis including all reports suggests that the restriction to HP reports had only a limited influence on the overall pattern of detected disproportionality signals.

High reporting rates have been identified for both clinical manifestations (cardiac failure, congestive heart failure, cardiogenic shock, and left ventricular failure), as well as for functional markers (decreased ejection fraction). The occurrence of signals across these different PTs reflects the range of cardiotoxicity-related events reported in the database, comprising the modifications of biomarkers like troponin, continuing with ventricular dysfunctionality (ejection fraction decreased), cardiomyopathy, and finally cardiac insufficiency and cardiogenic shock. The observed differences between doxorubicin and epirubicin can be expected, as epirubicin is the 4′- epimer of doxorubicin and has faster clearance, which is thought to contribute to lower myocardial exposure. The pharmacokinetic profile of epirubicin allows more extensive glucuronidation, exhibiting higher plasma clearance and a shorter mean residence time than doxorubicin. These characteristics have been proposed as factors that may influence systemic and myocardial exposure and are thought to contribute to its reduced cardiotoxicity compared with doxorubicin. Epirubicin may also involve inhibition of topoisomerase IIβ, oxidative stress, mitochondrial dysfunction, cell death, and ventricular remodeling [29].

No statistical differences were noted between doxorubicin and epirubicin for reporting “Acute pulmonary oedema”, “Heart failure with midrange ejection fraction”, “Heart failure with reduced ejection fraction”, “Myocardial fibrosis”, “Acute coronary syndrome”, and “Troponin I increased”. The finding might be explained by considering several possibilities: these rare events and the number of reports is low; furthermore, diagnosing these modifications requires imaging investigations or blood tests [1], taking a longer time to establish the diagnosis than direct patient observation. The possibility of preferential classification of cases under other, more general PTs (e.g., cardiac failure instead of heart failure with reduced ejection fraction) should also be considered [42].

The clinical interpretation of these findings should remain within the inherent limitations of spontaneous-reporting data. The observed reporting patterns and disproportionality signals highlight potential differences in the cardiovascular safety profiles of doxorubicin and epirubicin and underscore the clinical relevance of cardiovascular risk assessment and monitoring in patients receiving anthracycline therapy. However, these findings do not support preferential use of epirubicin over doxorubicin in patients at increased cardiovascular risk and should not be used alone to guide treatment selection. Rather, they are hypothesis-generating and may help inform future comparative studies evaluating cardiovascular outcomes and monitoring strategies. An integrated cardio-oncology approach remains relevant to the assessment and management of cardiovascular risk in patients receiving anthracyclines.

### Limitations of the Study

This study has several limitations associated with the use of data from spontaneous reporting systems, such as the EV database. Direct comparison with other spontaneous reporting systems, such as FAERS, was beyond the scope of this study because ICSRs may be reported to one or both databases, and the degree of overlap cannot be identified. Furthermore, differences in reporting practices, regulatory frameworks, and database characteristics may affect reporting patterns and should be considered when interpreting cross-database comparisons. First, these systems are susceptible to underreporting, selective reporting, and variations in the quality of clinical information [43]. Therefore, the number of reports cannot be considered representative of the actual incidence of cardiotoxic events in the population exposed to doxorubicin or epirubicin. Second, disproportionality analysis, including the use of ROR, identifies statistical associations between drugs and adverse events, but cannot demonstrate a causal relationship [44]. A signal of disproportionality should be interpreted as a pharmacovigilance signal requiring further assessment and not as an estimate of absolute risk or incidence of cardiotoxicity. Another potential limitation is notoriety bias, as the well-established cardiotoxicity of anthracyclines may increase clinicians’ awareness and, consequently, the likelihood of reporting cardiac adverse events. This may partly explain the stronger disproportionality signals observed for nonspecific terms such as “Cardiotoxicity” and “Cardiomyopathy” and may not necessarily reflect true differences in the underlying risk of cardiotoxicity between doxorubicin and epirubicin.

An important limitation is the lack of complete information on drug exposure and the absence of exposure denominators. In particular, the data from EV does not allow for the calculation of incidence rates and does not always provide sufficient information for an accurate assessment of the cumulative anthracycline dose, the dose administered per cycle, the total duration of treatment or the interval between doses. This is particularly relevant because the cardiotoxicity of anthracyclines may be influenced by cumulative exposure, and the risk of cardiac damage may vary depending on the total dose administered [45]. In addition, the differences in exposure between doxorubicin and epirubicin cannot be fully assessed based on the number of spontaneous reports. Also, information on pre-existing cardiovascular risk factors may be incomplete or absent. Moreover, the available data does not permit adequate adjustment for potential differences in patient population, treatment indications, cancer characteristics, or other clinical factors between reports involving doxorubicin or epirubicin. Consequently, direct comparisons of disproportionality between the two anthracyclines must be interpreted with caution. Another important factor is concomitant treatment and exposure to other potentially cardiotoxic therapies. Cancer patients may receive chest radiotherapy, other chemotherapy, targeted therapies or immunotherapies that can contribute to the development of cardiac dysfunction. In the absence of complete clinical data, the individual contribution of doxorubicin or epirubicin to the reported event cannot always be separated from the effect of other treatments [13]. Despite these limitations related to spontaneous pharmacovigilance data, the study provides a comparative assessment of reported cardiotoxicity signals associated with doxorubicin and epirubicin in a real-world pharmacovigilance setting and contributes to a better characterization of their cardiovascular safety profile, while providing a basis for formulating hypotheses that can be further investigated through prospective clinical and epidemiological studies.

## 5. Conclusions

This comparative pharmacovigilance analysis of EV reports identified a more pronounced pattern of reporting and disproportionality signals for selected cardiotoxicity-related adverse events reported for doxorubicin than for epirubicin. In the comparative disproportionality analysis, doxorubicin showed significantly higher reporting odds for several clinically relevant events, including cardiogenic shock, cardiac failure, decreased ejection fraction, cardiomyopathy, cardiotoxicity, acute myocardial infarction, myocardial infarction, and increased troponin.

These reporting patterns and disproportionality signals suggest potential differences in the cardiovascular safety profiles of doxorubicin and epirubicin that warrant further investigation in appropriately designed comparative studies. Nevertheless, cardiotoxic events were reported for both agents, highlighting the need for baseline cardiovascular assessment and continued monitoring during and after treatment.

Given the inherent limitations of spontaneous reporting systems, including underreporting, reporting bias, the absence of exposure denominators, and incomplete clinical information, these findings should be interpreted with caution. Disproportionality analyses identify reporting signals rather than incidence, comparative risk, or causal associations and do not permit conclusions regarding the preferential clinical use of one anthracycline over the other. Therefore, the observed reporting patterns should be regarded as hypothesis-generating and require confirmation in well-designed prospective pharmacoepidemiologic and clinical studies.

## Figures and Tables

**Table 1:** Characteristics of records associated with doxorubicin and epirubicin in EudraVigilance. EEA—European Economic Area.

	Doxorubicin	Epirubicin
	*n* (%)	*n* (%)
Total	52,257 (100.0%)	31,049 (100.0%)
Age category		
Not Specified	9904 (19.0%)	1380 (4.4%)
0–1 Month	69 (0.1%)	30 (0.1%)
2 Months–2 Years	554 (1.1%)	92 (0.3%)
3–11 Years	1997 (3.8%)	229 (0.7%)
12–17 Years	1691 (3.2%)	169 (0.5%)
18–64 Years	24,713 (47.3%)	23,849 (76.8%)
65–85 Years	12,869 (24.6%)	5249 (16.9%)
More than 85 Years	460 (0.9%)	51 (0.2%)
Sex		
Female	28,790 (55.1%)	26,550 (85.5%)
Male	18,876 (36.1%)	4044 (13.0%)
Not Specified	4591 (8.8%)	455 (1.5%)
Origin		
EEA	17,207 (32.9%)	6175 (19.9%)
NON-EEA	35,049 (67.1%)	24,874 (80.1%)
Not Specified	1 (0.0%)	0 (0.0%)
Reporter		
Healthcare Professional	49,815 (95.3%)	27,759 (89.4%)
Non-Healthcare Professional	2176 (4.2%)	3163 (10.2%)
Not Specified	266 (0.5%)	127 (0.4%)

**Table 2:** Distribution of cases by SOCs (number and rank).

Doxorubicin SOC	n	Rank	Epirubicin SOC	n
**Blood and lymphatic system disorders**	17,273	1	Blood and lymphatic system disorders	16,050
**General disorders and administration site conditions**	12,638	2	Investigations	9362
**Infections and infestations**	9334	3	Gastrointestinal disorders	4292
**Neoplasms benign, malignant and unspecified (incl. cysts and polyps)**	7998	4	General disorders and administration site conditions	4141
**Investigations**	6685	5	Hepatobiliary disorders	1676
**Gastrointestinal disorders**	6345	6	Cardiac disorders	1406
**Injury, poisoning and procedural complications**	6340	7	Skin and subcutaneous tissue disorders	1306
**Respiratory, thoracic and mediastinal disorders**	5457	8	Respiratory, thoracic and mediastinal disorders	1235
**Nervous system disorders**	4593	9	Nervous system disorders	1209
**Cardiac disorders**	4459	10	Neoplasms benign, malignant and unspecified (incl. cysts and polyps)	1178
**Skin and subcutaneous tissue disorders**	4183	11	Infections and infestations	1073
**Vascular disorders**	2243	12	Metabolism and nutrition disorders	1033
**Metabolism and nutrition disorders**	2149	13	Injury, poisoning and procedural complications	944
**Musculoskeletal and connective tissue disorders**	2015	14	Vascular disorders	572
**Hepatobiliary disorders**	1855	15	Musculoskeletal and connective tissue disorders	566
**Renal and urinary disorders**	1745	16	Renal and urinary disorders	350
**Immune system disorders**	1641	17	Psychiatric disorders	310
**Psychiatric disorders**	749	18	Immune system disorders	269
**Eye disorders**	498	19	Endocrine disorders	255
**Pregnancy, puerperium and perinatal conditions**	485	20	Eye disorders	153
**Endocrine disorders**	445	21	Reproductive system and breast disorders	118
**Congenital, familial and genetic disorders**	434	22	Pregnancy, puerperium and perinatal conditions	88
**Reproductive system and breast disorders**	360	23	Surgical and medical procedures	80
**Surgical and medical procedures**	349	24	Ear and labyrinth disorders	65
**Ear and labyrinth disorders**	222	25	Congenital, familial and genetic disorders	56
**Social circumstances**	90	26	Social circumstances	28
**Product issues**	70	27	Product issues	10

**Table 3:** Distribution of serious versus non-serious cases by SOCs (number and frequency).

RA by Seriousness	Doxorubicin			Epirubicin		
	Non- Serious n (%)	Not Specified n (%)	Serious n (%)	Non- Serious n (%)	Not Specified n (%)	Serious n (%)
Blood and lymphatic system disorders	318 (1.8%)	4 (0.0%)	16,951 (98.1%)	261 (1.6%)	28 (0.2%)	15,761 (98.2%)
Cardiac disorders	69 (1.5%)	2 (0.0%)	4388 (98.4%)	34 (2.4%)	9 (0.6%)	1363 (97.0%)
Congenital, familial and genetic disorders	11 (2.5%)	0 (0.0%)	423 (97.5%)	0 (0.0%)	0 (0.0%)	56 (100.0%)
Ear and labyrinth disorders	13 (5.9%)	0 (0.0%)	209 (94.1%)	18 (27.7%)	0 (0.0%)	47 (72.3%)
Endocrine disorders	25 (5.6%)	1 (0.2%)	419 (94.2%)	7 (2.7%)	0 (0.0%)	248 (97.3%)
Eye disorders	35 (7.0%)	0 (0.0%)	463 (93.0%)	34 (22.2%)	4 (2.6%)	115 (75.2%)
Gastrointestinal disorders	556 (8.8%)	6 (0.1%)	5783 (91.1%)	814 (19.0%)	8 (0.2%)	3470 (80.8%)
General disorders and administration site conditions	1040 (8.2%)	8 (0.1%)	11,590 (91.7%)	508 (12.3%)	23 (0.6%)	3610 (87.2%)
Hepatobiliary disorders	51 (2.7%)	0 (0.0%)	1804 (97.3%)	84 (5.0%)	1 (0.1%)	1591 (94.9%)
Immune system disorders	81 (4.9%)	3 (0.2%)	1,557 (94.9%)	10 (3.7%)	6 (2.2%)	253 (94.1%)
Infections and infestations	121 (1.3%)	4 (0.0%)	9209 (98.7%)	64 (6.0%)	10 (0.9%)	999 (93.1%)
Injury, poisoning and procedural complications	428 (6.8%)	2 (0.0%)	5910 (93.2%)	65 (6.9%)	1 (0.1%)	878 (93.0%)
Investigations	335 (5.0%)	3 (0.0%)	6347 (94.9%)	156 (1.7%)	3 (0.0%)	9203 (98.3%)
Metabolism and nutrition disorders	56 (2.6%)	2 (0.1%)	2091 (97.3%)	48 (4.6%)	2 (0.2%)	983 (95.2%)
Musculoskeletal and connective tissue disorders	276 (13.7%)	1 (0.0%)	1738 (86.3%)	111 (19.6%)	1 (0.2%)	454 (80.2%)
Neoplasms benign, malignant and unspecified (incl cysts and polyps)	46 (0.6%)	2 (0.0%)	7950 (99.4%)	19 (1.6%)	2 (0.2%)	1157 (98.2%)
Nervous system disorders	229 (5.0%)	8 (0.2%)	4356 (94.8%)	223 (18.4%)	5 (0.4%)	981 (81.1%)
Pregnancy, puerperium and perinatal conditions	21 (4.3%)	0 (0.0%)	464 (95.7%)	2 (2.3%)	0 (0.0%)	86 (97.7%)
Product issues	6 (8.6%)	0 (0.0%)	64 (91.4%)	2 (20.0%)	0 (0.0%)	8 (80.0%)
Psychiatric disorders	34 (4.5%)	0 (0.0%)	715 (95.5%)	25 (8.1%)	1 (0.3%)	284 (91.6%)
Renal and urinary disorders	30 (1.7%)	2 (0.1%)	1713 (98.2%)	24 (6.9%)	3 (0.9%)	323 (92.3%)
Reproductive system and breast disorders	32 (8.9%)	0 (0.0%)	328 (91.1%)	29 (24.6%)	0 (0.0%)	89 (75.4%)
Respiratory, thoracic and mediastinal disorders	196 (3.6%)	9 (0.2%)	5252 (96.2%)	83 (6.7%)	8 (0.6%)	1144 (92.6%)
Skin and subcutaneous tissue disorders	575 (13.7%)	13 (0.3%)	3595 (86.0%)	294 (22.5%)	10 (0.8%)	1002 (76.7%)
Social circumstances	7 (7.8%)	0 (0.0%)	83 (92.2%)	3 (10.7%)	0 (0.0%)	25 (89.3%)
Surgical and medical procedures	8 (2.3%)	0 (0.0%)	341 (97.7%)	6 (7.5%)	0 (0.0%)	74 (92.5%)
Vascular disorders	165 (7.4%)	7 (0.3%)	2071 (92.3%)	60 (10.5%)	12 (2.1%)	500 (87.4%)

## Data Availability

Publicly available datasets were analyzed in this study. This data can be found here: https://www.adrreports.eu/ (accessed on 14 July 2026).

## Supplementary Materials

The following supporting information can be downloaded at: https://www.mdpi.com/article/10.3390/jcm15176734/s1, Table S1: Preferred terms selected for evaluation of cardiotoxicity; Table S2: Frequency of ADRs reported for each PT of interest; Table S3: Number of reports by clinical outcomes; Table S4: Reporting odds ratios (RORs) and 95% confidence intervals (CIs) values for ADRs reported by HP; Table S5: Reporting odds ratios (RORs) and 95% confidence intervals (CIs) for the overall EudraVigilance dataset (all reporters).

## Abbreviations

The following abbreviations are used in this manuscript:

ADR	Adverse drug reaction
BNP	B-type natriuretic peptide
CI	Confidence interval
EEA	European Economic Area
EMA	European Medicines Agency
EV	EudraVigilance
FAERS	FDA Adverse Event Reporting System
HP	Healthcare professionals
ICSR	Individual Case Safety Reports
MedDRA	Medical Dictionary for Regulatory Activities
PT	Preferred term
ROR	Reporting odds ratio
SmPC	Summary of Product Characteristics
SMQ	Standardized MedDRA Queries
SOC	System organ class
