# Supplementary material for: Comparative Pharmacovigilance Analysis of Selected Cardiotoxicity Signals Associated with Doxorubicin and Epirubicin Based on EudraVigilance Data
Source: J Clin Med. 2026 Aug 30;15(17):6734. doi: 10.3390/jcm15176734 (PMC13565764; doi:10.3390/jcm15176734)
Supplement: Supplementary file 1 [file jcm-15-06734-s001.zip › jcm-4478280-supplementary.pdf]

Supplementary Material

Table S1. Preferred terms selected for evaluation of cardiotoxicity. PT – Preferred Term, SMQ – Standardized MedDRA Queries

SMQ_code	SMQ	PT code	PT
20000004	Cardiac failure	10063081	Acute left ventricular failure
20000004	Cardiac failure	10001029	Acute pulmonary oedema
20000004	Cardiac failure	10007554	Cardiac failure
20000004	Cardiac failure	10007556	Cardiac failure acute
20000004	Cardiac failure	10007558	Cardiac failure chronic
20000004	Cardiac failure	10007559	Cardiac failure congestive
20000004	Cardiac failure	10007560	Cardiac failure high output
20000004	Cardiac failure	10007625	Cardiogenic shock
20000004	Cardiac failure	10051093	Cardiopulmonary failure
20000004	Cardiac failure	10068230	Cardiorenal syndrome
20000004	Cardiac failure	10063083	Chronic left ventricular failure
20000004	Cardiac failure	10084058	Congestive hepatopathy
20000004	Cardiac failure	10010968	Cor pulmonale
20000004	Cardiac failure	10050528	Ejection fraction decreased
20000004	Cardiac failure	10086366	Heart failure with midrange ejection fraction
20000004	Cardiac failure	10076396	Heart failure with preserved ejection fraction
20000004	Cardiac failure	10078289	Heart failure with reduced ejection fraction
20000004	Cardiac failure	10051448	Hepatojugular reflux
20000004	Cardiac failure	10024119	Left ventricular failure
20000004	Cardiac failure	10024899	Low cardiac output syndrome
20000004	Cardiac failure	10049780	Neonatal cardiac failure
20000004	Cardiac failure	10039163	Right ventricular failure
20000004	Cardiac failure	10060953	Ventricular failure
20000150	Cardiomyopathy	10007509	Cardiac amyloidosis

20000150	Cardiomyopathy	10007572	Cardiac hypertrophy
20000150	Cardiomyopathy	10007604	Cardiac sarcoidosis
20000150	Cardiomyopathy	10007636	Cardiomyopathy
20000150	Cardiomyopathy	10048377	Cardiomyopathy acute
20000150	Cardiomyopathy	10050111	Cardiomyopathy neonatal
20000150	Cardiomyopathy	10048610	Cardiotoxicity
20000150	Cardiomyopathy	10056419	Dilated cardiomyopathy
20000150	Cardiomyopathy	10014961	Eosinophilic myocarditis
20000150	Cardiomyopathy	10048858	Ischaemic cardiomyopathy
20000150	Cardiomyopathy	10028594	Myocardial fibrosis
20000150	Cardiomyopathy	10049813	Non-obstructive cardiomyopathy
20000150	Cardiomyopathy	10038748	Restrictive cardiomyopathy
20000150	Cardiomyopathy	10066286	Stress cardiomyopathy
20000150	Cardiomyopathy	10083657	Toxic cardiomyopathy
20000047	Myocardial infarction	10051592	Acute coronary syndrome
20000047	Myocardial infarction	10000891	Acute myocardial infarction
20000047	Myocardial infarction	10002388	Angina unstable
20000047	Myocardial infarction	10011086	Coronary artery occlusion
20000047	Myocardial infarction	10011091	Coronary artery thrombosis
20000047	Myocardial infarction	10069167	Kounis syndrome
20000047	Myocardial infarction	10028596	Myocardial infarction
20000047	Myocardial infarction	10028602	Myocardial necrosis
20000047	Myocardial infarction	10072186	Myocardial stunning
20000047	Myocardial infarction	10049768	Silent myocardial infarction
20000047	Myocardial infarction	10058268	Troponin I increased
20000047	Myocardial infarction	10058267	Troponin increased
20000047	Myocardial infarction	10058269	Troponin T increased

Table S2. Frequency of ADRs reported for each PT of interest

SMQ code	SMQ	PT code	PT	Total cases reported for epirubicin	Total cases reported for doxorubicin
**20000004**	Cardiac failure	10063081	Acute left ventricular failure	0	11
**20000004**	Cardiac failure	10001029	Acute pulmonary oedema	16	50
**20000004**	Cardiac failure	10007554	Cardiac failure	352	923
**20000004**	Cardiac failure	10007556	Cardiac failure acute	33	99
**20000004**	Cardiac failure	10007558	Cardiac failure chronic	12	41
**20000004**	Cardiac failure	10007559	Cardiac failure congestive	66	284
**20000004**	Cardiac failure	10007560	Cardiac failure high output	0	1
**20000004**	Cardiac failure	10007625	Cardiogenic shock	15	108
**20000004**	Cardiac failure	10051093	Cardiopulmonary failure	1	8
**20000004**	Cardiac failure	10068230	Cardiorenal syndrome	0	4
**20000004**	Cardiac failure	10063083	Chronic left ventricular failure	0	1
**20000004**	Cardiac failure	10084058	Congestive hepatopathy	3	5
**20000004**	Cardiac failure	10010968	Cor pulmonale	0	1
**20000004**	Cardiac failure	10050528	Ejection fraction decreased	163	530
**20000004**	Cardiac failure	10086366	Heart failure with midrange ejection fraction	6	5
**20000004**	Cardiac failure	10076396	Heart failure with preserved ejection fraction	1	1
**20000004**	Cardiac failure	10078289	Heart failure with reduced ejection fraction	22	53
**20000004**	Cardiac failure	10051448	Hepatojugular reflux	1	3
**20000004**	Cardiac failure	10024119	Left ventricular failure	17	52
**20000004**	Cardiac failure	10024899	Low cardiac output syndrome	1	3
**20000004**	Cardiac failure	10049780	Neonatal cardiac failure	0	1
**20000004**	Cardiac failure	10039163	Right ventricular failure	0	12
**20000004**	Cardiac failure	10060953	Ventricular failure	1	6
**20000150**	Cardiomyopathy	10007509	Cardiac amyloidosis	1	4
**20000150**	Cardiomyopathy	10007572	Cardiac hypertrophy	1	5

**20000150**	Cardiomyopathy	10007604	Cardiac sarcoidosis	1	1
**20000150**	Cardiomyopathy	10007636	Cardiomyopathy	147	689
**20000150**	Cardiomyopathy	10048377	Cardiomyopathy acute	0	12
**20000150**	Cardiomyopathy	10050111	Cardiomyopathy neonatal	0	1
**20000150**	Cardiomyopathy	10048610	Cardiotoxicity	155	660
**20000150**	Cardiomyopathy	10056419	Dilated cardiomyopathy	65	182
**20000150**	Cardiomyopathy	10014961	Eosinophilic myocarditis	0	1
**20000150**	Cardiomyopathy	10048858	Ischaemic cardiomyopathy	1	2
**20000150**	Cardiomyopathy	10028594	Myocardial fibrosis	5	13
**20000150**	Cardiomyopathy	10049813	Non-obstructive cardiomyopathy	0	2
**20000150**	Cardiomyopathy	10038748	Restrictive cardiomyopathy	1	6
**20000150**	Cardiomyopathy	10066286	Stress cardiomyopathy	1	24
**20000150**	Cardiomyopathy	10083657	Toxic cardiomyopathy	6	36
**20000047**	Myocardial infarction	10051592	Acute coronary syndrome	9	26
**20000047**	Myocardial infarction	10000891	Acute myocardial infarction	20	77
**20000047**	Myocardial infarction	10002388	Angina unstable	0	11
**20000047**	Myocardial infarction	10011086	Coronary artery occlusion	1	2
**20000047**	Myocardial infarction	10011091	Coronary artery thrombosis	1	4
**20000047**	Myocardial infarction	10069167	Kounis syndrome	2	2
**20000047**	Myocardial infarction	10028596	Myocardial infarction	31	104
**20000047**	Myocardial infarction	10028602	Myocardial necrosis	0	1
**20000047**	Myocardial infarction	10072186	Myocardial stunning	0	1
**20000047**	Myocardial infarction	10049768	Silent myocardial infarction	0	2
**20000047**	Myocardial infarction	10058268	Troponin I increased	8	16
**20000047**	Myocardial infarction	10058267	Troponin increased	21	64
**20000047**	Myocardial infarction	10058269	Troponin T increased	3	14

Table S3. Number of reports by clinical outcomes

SMQ	PT	Doxorubicin								Epirubicin							
		**Total ADRs**	**Fatal**	**Not Recovered/Not Resolved**	**Not Specifed**	**Recovered/Resolved**	**Recovered/Resolved with Sequelae**	**Recovering/Resolving**	**Unknown**	**Total ADRs**	**Fatal**	**Not Recovered/ Not Resolved**	**Not Specified**	**Recovered/Resolved**	**Recovered/Resolved with Sequelae**	**Recovering/Resolving**	**Unknown**
**Cardiac failure**	Acute left ventricular failure	11	0	0	0	5	0	3	3	0	0	0	0	0	0	0	0
	Acute pulmonary oedema	50	4	6	1	24	0	8	7	16	0	2	0	6	3	2	3
	Cardiac failure	923	134	141	9	146	36	172	285	352	26	84	2	52	21	95	72
	Cardiac failure acute	99	13	7	0	15	4	30	30	33	3	3	0	11	3	10	3
	Cardiac failure chronic	41	1	4	0	8	1	13	14	12	0	6	0	3	0	2	1
	Cardiac failure congestive	284	36	24	7	49	4	52	112	66	3	10	1	14	0	24	14
	Cardiac failure high output	1	0	0	1	0	0	0	0	0	0	0	0	0	0	0	0
	Cardiogenic shock	108	31	11	2	30	3	11	20	15	5	1	0	4	0	4	1
	Cardiopulmonary failure	8	6	1	0	0	0	0	1	1	0	0	0	1	0	0	0
	Cardiorenal syndrome	4	2	0	0	1	0	0	1	0	0	0	0	0	0	0	0
	Chronic left ventricular failure	1	0	0	0	0	0	1	0	0	0	0	0	0	0	0	0
	Congestive hepatopathy	5	2	1	0	0	0	0	2	3	1	0	0	1	0	1	0
	Cor pulmonale	1	1	0	0	0	0	0	0	0	0	0	0	0	0	0	0
	Ejection fraction decreased	530	17	122	7	78	6	67	233	163	2	53	1	37	2	23	45
	Heart failure with midrange ejection fraction	5	0	3	0	0	0	1	1	6	0	3	0	0	0	3	0

	Heart failure with preserved ejection fraction	1	0	0	0	0	0	1	0	1	0	0	0	0	0	1	0
	Heart failure with reduced ejection fraction	53	1	17	0	8	0	20	7	22	0	18	0	0	1	2	1
	Hepatojugular reflux	3	0	0	0	0	0	0	3	1	0	0	0	0	0	0	1
	Left ventricular failure	52	2	13	2	10	2	7	16	17	1	7	0	2	0	4	3
	Low cardiac output syndrome	3	1	0	0	0	0	1	1	1	0	0	0	0	0	1	0
	Neonatal cardiac failure	1	1	0	0	0	0	0	0	0	0	0	0	0	0	0	0
	Right ventricular failure	12	1	2	0	2	1	2	4	0	0	0	0	0	0	0	0
	Ventricular failure	6	1	0	0	4	0	0	1	1	0	0	0	0	0	1	0
**Cardiomyopathy**	Cardiac amyloidosis	4	0	2	0	1	0	0	1	1	0	1	0	0	0	0	0
	Cardiac hypertrophy	5	0	1	0	0	0	1	3	1	0	1	0	0	0	0	0
	Cardiac sarcoidosis	1	0	0	0	0	0	0	1	1	0	0	0	0	0	0	1
	Cardiomyopathy	689	44	69	6	113	17	152	288	147	5	30	0	24	5	34	49
	Cardiomyopathy acute	12	2	1	0	2	0	5	2	0	0	0	0	0	0	0	0
	Cardiomyopathy neonatal	1	0	0	0	1	0	0	0	0	0	0	0	0	0	0	0
	Cardiotoxicity	660	27	40	3	103	5	65	417	155	3	15	0	23	3	39	72
	Dilated cardiomyopathy	182	13	40	2	15	12	31	69	65	7	37	0	2	8	5	6
	Eosinophilic myocarditis	1	1	0	0	0	0	0	0	0	0	0	0	0	0	0	0
	Ischaemic cardiomyopathy	2	0	1	0	0	0	0	1	1	0	1	0	0	0	0	0
	Myocardial fibrosis	13	2	1	0	4	0	1	5	5	0	1	0	1	0	1	2
	Non-obstructive cardiomyopathy	2	0	2	0	0	0	0	0	0	0	0	0	0	0	0	0
	Restrictive cardiomyopathy	6	2	1	0	0	0	0	3	1	0	0	0	1	0	0	0

	Stress cardiomyopathy	24	2	1	0	11	0	4	6	1	0	0	0	1	0	0	0
	Toxic cardiomyopathy	36	6	4	0	4	1	13	8	6	2	2	0	1	0	1	0
**Myocardial infarction**	Acute coronary syndrome	26	2	1	0	13	2	4	4	9	0	0	0	6	1	0	2
	Acute myocardial infarction	77	19	7	2	13	4	8	24	20	3	3	0	2	1	3	8
	Angina unstable	11	0	8	0	2	0	0	1	0	0	0	0	0	0	0	0
	Coronary artery occlusion	2	0	0	0	1	0	0	1	1	0	0	0	0	0	0	1
	Coronary artery thrombosis	4	0	0	0	1	0	2	1	1	0	0	0	1	0	0	0
	Kounis syndrome	2	0	0	0	2	0	0	0	2	0	0	0	2	0	0	0
	Myocardial infarction	104	28	2	3	23	4	9	35	31	6	2	1	4	2	2	14
	Myocardial necrosis	1	1	0	0	0	0	0	0	0	0	0	0	0	0	0	0
	Myocardial stunning	1	0	0	0	0	0	1	0	0	0	0	0	0	0	0	0
	Silent myocardial infarction	2	0	0	0	0	0	0	2	0	0	0	0	0	0	0	0
	Troponin I increased	16	0	4	0	4	0	2	6	8	0	0	0	1	0	4	3
	Troponin increased	64	0	20	0	15	0	12	17	21	0	6	0	5	0	7	3
	Troponin T increased	14	0	2	0	3	1	3	5	3	0	0	0	1	0	1	1

Table S4. Reporting odds ratios (RORs) and 95% confidence intervals (CIs) values for ADRs reported by HP

SMQ	PT	ADRs of interest reported for doxorubicin	Other ADRs reported for doxorubicin	ADRs of interest reported for epirubicin	Other ADRs reported for epirubicin	ROR	95% CI minim	95% CI maxim	p-value
**Cardiac failure**	Acute pulmonary oedema	50	49,765	16	27,743	1.74	0.99	3.06	P = 0.0534
	Cardiac failure	881	48,934	331	27,428	1.49	1.31	1.69	P < 0.0001
	Cardiac failure acute	98	49,717	33	27,726	1.66	1.12	2.46	P = 0.0123
	Cardiac failure chronic	36	49,779	9	27,750	2.23	1.07	4.63	P = 0.0314
	Cardiac failure congestive	268	49,547	60	27,699	2.50	1.89	3.30	P < 0.0001
	Cardiogenic shock	107	49,708	15	27,744	3.98	2.32	6.84	P < 0.0001
	Ejection fraction decreased	508	49,307	157	27,602	1.81	1.51	2.17	P < 0.0001
	Heart failure with midrange ejection fraction	5	49,810	6	27,753	0.46	0.14	1.52	P = 0.2052
	Heart failure with reduced ejection fraction	49	49,766	19	27,740	1.44	0.85	2.44	P = 0.1795
	Left ventricular failure	50	49,765	14	27,745	1.99	1.10	3.60	P = 0.0228
**Cardiomyopathy**	Cardiomyopathy	667	49,148	140	27,619	2.68	2.23	3.21	P < 0.0001
	Cardiotoxicity	640	49,175	135	27,624	2.66	2.21	3.21	P < 0.0001
	Dilated cardiomyopathy	181	49,634	61	27,698	1.66	1.24	2.21	P = 0.0007
	Myocardial fibrosis	13	49,802	5	27,754	1.45	0.52	4.06	P = 0.4810
	Toxic cardiomyopathy	35	49,780	6	27,753	3.25	1.37	7.73	P = 0.0076
**Myocardial infarction**	Acute coronary syndrome	26	49,789	9	27,750	1.61	0.75	3.44	P = 0.2182
	Acute myocardial infarction	72	49,743	20	27,739	2.01	1.22	3.30	P = 0.0058
	Myocardial infarction	98	49,717	29	27,730	1.88	1.25	2.85	P = 0.0027
	Troponin I increased	15	49,800	8	27,751	1.04	0.44	2.46	P = 0.9202
	Troponin increased	63	49,752	21	27,738	1.67	1.02	2.74	P = 0.0413

Table S5. Reporting odds ratios (RORs) and 95% confidence intervals (CIs) for the overall EudraVigilance dataset (all reporters).

SMQ	PT	ADRs of interest reported for doxorubicin	Other ADRs reported for doxorubicin	ADRs of interest reported for epirubicin	Other ADRs reported for epirubicin	ROR	95% CI minim	95% CI maxim	p-value
**Cardiac failure**	Acute pulmonary oedema	50	52,207	16	31,033	1.86	1.06	3.26	P = 0.0311
	Cardiac failure	923	51,334	352	30,697	1.57	1.39	1.77	P < 0.0001
	Cardiac failure acute	99	52,158	33	31,016	1.78	1.20	2.65	P = 0.0040
	Cardiac failure chronic	41	52,216	12	31,037	2.03	1.07	3.86	P = 0.0309
	Cardiac failure congestive	284	51,973	66	30,983	2.57	1.96	3.35	P < 0.0001
	Cardiogenic shock	108	52,149	15	31,034	4.28	2.50	7.35	P < 0.0001
	Ejection fraction decreased	530	51,727	163	30,886	1.94	1.63	2.32	P < 0.0001
	Heart failure with midrange ejection fraction	5	52,252	6	31,043	0.50	0.15	1.62	P = 0.2457
	Heart failure with reduced ejection fraction	53	52,204	22	31,027	1.43	0.87	2.35	P = 0.1571
	Left ventricular failure	52	52,205	17	31,032	1.82	1.05	3.14	P = 0.0324
**Cardiomyopathy**	Cardiomyopathy	689	51,568	147	30,902	2.81	2.35	3.36	P < 0.0001
	Cardiotoxicity	660	51,597	155	30,894	2.55	2.14	3.04	P < 0.0001
	Dilated cardiomyopathy	182	52,075	65	30,984	1.67	1.25	2.21	P = 0.0004
	Myocardial fibrosis	13	52,244	5	31,044	1.54	0.55	4.33	P = 0.4085
	Toxic cardiomyopathy	36	52,221	6	31,043	3.57	1.50	8.47	P = 0.0039
**Myocardial infarction**	Acute coronary syndrome	26	52,231	9	31,040	1.72	0.80	3.66	P = 0.1623
	Acute myocardial infarction	77	52,180	20	31,029	2.29	1.40	3.74	P = 0.0010
	Myocardial infarction	104	52,153	31	31,018	2.00	1.34	2.98	P = 0.0007
	Troponin I increased	16	52,241	8	31,041	1.19	0.51	2.78	P = 0.6902
	Troponin increased	64	52,193	21	31,028	1.81	1.11	2.97	P = 0.0182
